# Neonatal Congenital Hyperinsulinism: A Case-Based Contribution to the Understanding of a Rare Disorder

**DOI:** 10.7759/cureus.89272

**Published:** 2025-08-03

**Authors:** Fouad Khalil El Ouadghiri, Anass Ayyad, Sahar Messaoudi, Rim Amrani

**Affiliations:** 1 Department of Neonatology, Faculty of Medicine and Pharmacy, Mohammed First University, Oujda, MAR; 2 Department of Neonatology, Mohammed VI University Hospital Center, Oujda, MAR; 3 Mother and Child Health Laboratory, Faculty of Medicine and Pharmacy, Mohammed First University, Oujda, MAR

**Keywords:** congenital hyperinsulinism, diazoxide, genetic mutations, inappropriate insulin secretion, neonatal hypoglycemia

## Abstract

Congenital hyperinsulinism (CHI) is a rare but significant cause of persistent neonatal hypoglycemia (NH), associated with a high risk of neurological complications if not promptly treated. This condition is characterized by inappropriate insulin secretion, often of genetic origin, independent of blood glucose levels.

We report the case of a male macrosomic newborn admitted on the second day of life for respiratory distress, generalized seizures, and severe hypoglycemia (1.4 mmol/L) unresponsive to intravenous glucose therapy. Laboratory investigations revealed elevated insulin and C-peptide levels, absence of ketone bodies, and a positive response to the glucagon stimulation test. Echocardiography showed hypertrophic cardiomyopathy without functional impairment. Due to limited resources, neither [18F]-fluoro-L-DOPA PET imaging nor genetic testing could be performed. Treatment with a combination of diazoxide and octreotide led to partial improvement, but the clinical course was unfavorable, with the infant dying at four months of age due to sepsis.

This case highlights the diagnostic and therapeutic challenges of CHI in resource-limited settings. Through this clinical observation and a review of the literature, we emphasize the importance of a rigorous diagnostic approach and early, multidisciplinary, and tailored management to reduce the morbidity and mortality associated with this rare condition.

## Introduction

Neonatal hypoglycemia (NH) is one of the most frequently encountered metabolic abnormalities in the neonatal period. It represents a major cause of morbidity in newborns and infants, particularly when it is not promptly recognized or appropriately managed. While transient forms of hypoglycemia are the most common and usually benign, persistent or recurrent hypoglycemia can lead to serious complications. Among the potential etiologies, congenital hyperinsulinism (CHI), although rare, is recognized as the most frequent and severe cause of persistent hypoglycemia during the neonatal period and early infancy [1]. CHI is a heterogeneous condition, both clinically and at the histological and molecular levels. Its incidence is estimated at one in 35,000 to one in 40,000 live births in the general population, but it can be as high as one in 2,500 in populations with high rates of consanguinity [2]. CHI is characterized by inappropriate insulin secretion, leading to excessive insulin levels irrespective of blood glucose concentration. Without early diagnosis and treatment, CHI can cause irreversible neurological sequelae, underscoring the importance of timely screening and appropriate management.

In this article, we report the case of a neonate with severe CHI, admitted for respiratory distress and generalized seizures associated with profound hypoglycemia, and we describe the therapeutic interventions undertaken. Using this clinical case as a foundation, our objective is to provide an updated overview of the clinical, diagnostic, and therapeutic aspects of CHI. Based on a selected review of the scientific literature, this article aims to offer clinicians, pediatricians, and neonatologists a clear and comprehensive synthesis of the current understanding of this rare but potentially life-threatening endocrine disorder.

## Case presentation

We report the case of a male neonate born to consanguineous parents (third-degree relatives). The 23-year-old mother had no significant medical history, including no diabetes or recent medication or toxic exposure. The pregnancy was well monitored and carried to term. Delivery was performed by cesarean section due to suspected fetal macrosomia. Apgar scores were 10/10 at birth, and there was no evidence of prenatal infection.

The newborn was admitted to the neonatal intensive care unit on the second day of life for respiratory distress, repeated generalized seizures, and severe hypoglycemia.

The initial clinical manifestations appeared at 30 hours of life and included moderate respiratory distress (Silverman score: 2-3/10), generalized seizures, and severe hypoglycemia with a blood glucose level of 1.4 mmol/L (equivalent to 25 mg/dL), refractory to intravenous glucose correction.

**Figure 1:**
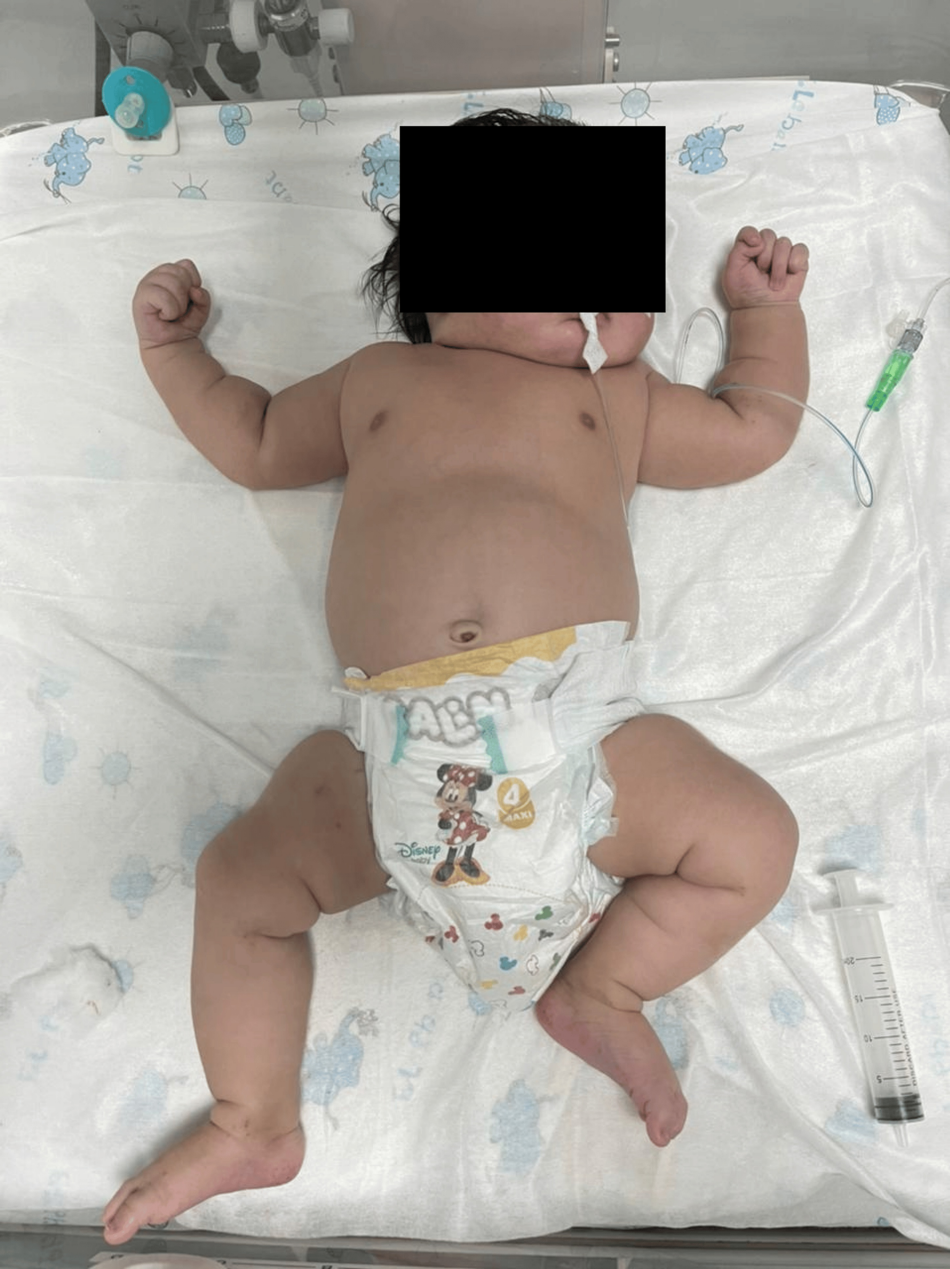
Newborn presenting with neonatal macrosomia secondary to CHI. Notable findings include abundant subcutaneous fat, a prominent abdomen, and relative hypotonia. CHI, congenital hyperinsulinism

On physical examination, the infant appeared subicteric with a pink background and was hypotonic but reactive. The sucking reflex was weak, while other primitive reflexes were present. Anthropometric measurements at birth indicated macrosomia: birth weight 5.5 kg, length 59 cm, and head circumference 44 cm, all above the 97th percentile (Figure 1). Respiratory assessment revealed oxygen saturation of 88% in room air. No hepatomegaly or micropenis was noted. The remainder of the physical examination was unremarkable. Metabolically, the patient experienced multiple episodes of hypoglycemia despite adequate intravenous glucose infusion and frequent feedings.

An initial infectious workup was performed. The complete blood count was within normal limits for age, but C-reactive protein (CRP) was elevated at 30 mg/L. A lumbar puncture was performed due to suspicion of central nervous system infection and returned negative.

The diagnosis of CHI was established based on persistent severe hypoglycemia, inappropriately elevated insulin and C-peptide levels during hypoglycemia, absence of ketogenesis, a positive glucagon stimulation test, and exclusion of other causes of NH. All the laboratory tests supporting the diagnosis are listed in Table 1.

**Table 1:** Laboratory findings during the hypoglycemic episode. GH, growth hormone

Parameter	Result	Reference Range	Interpretation
Blood glucose	1.4 mmol/L	>2.6 mmol/L	Severe hypoglycemia
C-peptide	4.92 ng/mL	0.5-2.0 ng/mL	Elevated
Insulin	32.20 µU/mL	<2-3 µU/mL (during hypoglycemia)	Elevated
Urinary ketone bodies	Absent	Present in physiological hypoglycemia	Consistent with hyperinsulinism
Glucagon stimulation test		Expected rise in blood glucose	Consistent with hyperinsulinism
GH	11.07 mU/L	>5 mU/L	Normal
IGF-1	180 ng/mL	Variable according to age	Normal
TSH	21.8 pmol/L	10-30 pmol/L (newborn)	Normal
Cortisol	Normal	>18 µg/dL	Normal
17-OH-progestérone	1.2 ng/mL	<3 ng/mL	Normal
Ammonia	71.17 µg/dL	<100 µg/dL	Normal
Lactate	3.16 mmol/L	0.5-2.2 mmol/L	Moderately elevated
Liver function tests	Normal		No abnormalities detected

Echocardiography revealed left ventricular hypertrophic cardiomyopathy without functional impairment. Morphological assessment by [18F]-fluoro-L-DOPA PET scan could not be performed due to its unavailability at our facility. Genetic testing was not conducted, as it is only accessible through private laboratories at the family’s expense.

Initial treatment consisted of a glucose infusion at 10 mg/kg/min, combined with feeding using maternal breast milk and premature infant formula. Due to persistent hypoglycemic episodes, diazoxide (17 mg/kg/day) was initiated in combination with octreotide (20 µg/kg/day). This led to a significant reduction in the frequency of hypoglycemia (down to one episode per day or every two days), but hypertrichosis appeared after one month of treatment.

Neurologically, initial anticonvulsant therapy was based on phenobarbital (3 mg/kg/day). At four months of age, the infant reportedly developed clinical signs consistent with sepsis, for which he was admitted to the emergency department of a local hospital. The clinical course rapidly worsened, resulting in the infant’s death.

## Discussion

NH is a common metabolic emergency during the neonatal period. Although its definition remains subject to debate, it is generally accepted as a plasma glucose concentration below 30 mg/dL (1.65 mmol/L) within the first 24 hours of life and below 45 mg/dL (2.5 mmol/L) thereafter. Hypoglycemia occurring in a newborn without identifiable risk factors, or presenting with unusual severity or persistence, should prompt suspicion of an underlying metabolic or endocrine disorder [1].

Pathophysiologically, hypoglycemia is often related to excessive insulin secretion, which stimulates glucose uptake by insulin-dependent tissues (skeletal muscle and adipose tissue) and inhibits hepatic glucose production via glycogenolysis and gluconeogenesis. Insulin also suppresses lipolysis and ketogenesis, thereby preventing the utilization of free fatty acids and the production of ketone bodies, depriving the brain of alternative energy sources. This mechanism explains the non-ketotic nature of hypoglycemia observed in the context of hyperinsulinism [3].

CHI represents the most frequent and severe cause of persistent hypoglycemia during the neonatal period. This rare disorder is caused by inactivating mutations affecting the regulation of the ATP-sensitive potassium (KATP) channel in pancreatic β-cells [4]. The channel consists of two main subunits: Kir6.2, which forms the channel pore, and SUR1 (sulfonylurea receptor), responsible for regulation. Inactivation of these subunits prevents channel opening, resulting in chronic membrane depolarization, persistent intracellular calcium influx, and inappropriate insulin secretion.

Two distinct histological lesions have been described in CHI, aside from the rare pancreatic adenomas. These are the diffuse form, which is the most common (approximately 60% of cases), and the focal form, accounting for nearly 40% of cases [5]. The diffuse form is characterized by generalized hyperplasia of pancreatic β-cells, with no histologically normal areas. Conversely, the focal form involves localized proliferation of hyperfunctional islets of Langerhans within an otherwise normal pancreatic parenchyma [5]. This morphological distinction is critical for management, particularly surgical intervention, and closely correlates with the underlying genetic etiology.

At the molecular level, CHI is predominantly caused by mutations affecting the KATP channel composed of two subunits: Kir6.2 (encoded by KCNJ11), forming the channel pore, and SUR1 (encoded by ABCC8), the regulatory subunit [6,7]. Mutations in these genes are responsible for the two main histological phenotypes of CHI. The diffuse form is generally associated with bi-allelic recessive inheritance, whereas the focal form classically results from paternal monoallelic transmission of a pathogenic variant combined with somatic loss of the maternal allele at locus 11p15.1 [8]. This localized somatic uniparental disomy explains the circumscribed nature of the histological abnormality. Beyond ABCC8 and KCNJ11, other genes have been implicated in persistent forms of CHI, including GLUD1, GCK, HADH, SLC16A1, HNF4A, HNF1A, and UCP2 [1]. These genetic anomalies, inherited through various modes (recessive, dominant, X-linked, or sporadic), influence clinical presentation, severity, and treatment response. Genetic diagnosis plays a central role not only in confirming the etiology of CHI but also in guiding therapeutic strategy, especially in cases of diazoxide resistance, which is the first-line treatment. Identification of two pathogenic variants in trans or a single dominant variant suggests a diffuse form, whereas a single paternal-recessive mutation strongly indicates a focal form, with a diagnostic sensitivity close to 97% [9]. In atypical presentations, comprehensive genomic sequencing is recommended.

The chromosomal region 11p15, which harbors the ABCC8 and KCNJ11 genes, is also involved in several rare genetic syndromes associated with CHI, most notably Beckwith-Wiedemann syndrome (BWS) [10]. Other syndromes, such as Kabuki, Sotos, or Turner syndrome, have also been reported [9,10]. To date, more than 28 syndromes have been described in association with CHI, although the precise mechanisms underlying insulin secretion dysregulation remain unclear in most cases. In many of these syndromes, the association with CHI is inconsistent, often transient, and does not directly involve the genes typically associated with CHI. Diagnosis may be further complicated because CHI symptoms can precede the appearance of characteristic dysmorphic features or because these features are subtle or nonspecific [9].

A particular form of CHI is linked to activating mutations in the GLUD1 gene on chromosome 10, responsible for the HI/hyperammonemia syndrome [11]. These mutations impair the regulation of amino acid-induced insulin secretion, leading to leucine-sensitive hypoglycemia associated with moderate hyperammonemia [8]. These forms are generally responsive to diazoxide.

CHI primarily manifests in the neonatal period, with early-onset hypoglycemia (before 72 hours of life), profound hypoglycemia (<2 mmol/L) occurring unpredictably in both fasting and postprandial states, and persisting despite adequate glucose intake. These episodes are non-ketotic, which is a major distinguishing feature [1]. The clinical presentation of CHI is heterogeneous. It may range from subtle signs such as pallor, hypotonia, or lethargy to more severe symptoms, including altered consciousness, seizures, coma, and cardiopulmonary manifestations such as cyanosis, apnea, hypothermia, or heart failure [12,13]. Hypertrophic cardiomyopathy or hepatomegaly may also be observed in some patients, likely related to the anabolic effects of fetal hyperinsulinism [2]. Additionally, facial dysmorphism, characterized by coarse facial features, a short columella, broad nose, and thin upper lip, may be noted in certain syndromic contexts [14].

From a biochemical standpoint, diagnosis is based on samples taken during a hypoglycemic episode (blood glucose <2.6 mmol/L). These analyses typically reveal inappropriate insulin levels, often exceeding 10 µIU/mL, as well as detectable C-peptide levels, confirming endogenous hyperinsulinism. This is accompanied by hypo-ketonemia and decreased free fatty acids, a requirement for high glucose infusion rates often exceeding 8 to 10 mg/kg/min to maintain blood glucose above 3 mmol/L, and a positive glycemic response to glucagon testing, reflecting mobilization of hepatic glycogen stores normally inhibited by insulin. These criteria align with those defined by Stanley and Baker [15]: hyperinsulinemia, hypo-ketonemia, low free fatty acids, and a hyperglycemic response to glucagon. Insulin is not always detectable during hypoglycemia; measurement of C-peptide, which has a longer half-life, can enhance diagnostic accuracy. There is no direct correlation between the severity of hypoglycemia and circulating insulin concentration.

Conventional morphological imaging modalities, such as ultrasound, computed tomography (CT), and magnetic resonance imaging (MRI), do not allow positive diagnosis or localization of lesions in CHI [5]. [18F]-fluoro-L-DOPA PET/CT is currently the gold standard for functional imaging. It enables visualization of a single focal hyperuptake in approximately 75% of focal CHI cases, guiding targeted surgical resection. This technique can be combined with CT angiography for improved anatomical precision. However, its performance is limited in diffuse forms, where uptake is homogeneous throughout the pancreas [16]. In certain specialized centers, transhepatic catheterization with sequential pancreatic venous sampling may be used. This procedure measures insulin and C-peptide concentrations in different pancreatic venous branches. A localized increase identifies a hypersecreting area. Nevertheless, this invasive and technically demanding procedure is no longer routinely used, especially where PET/CT access is available. Therefore, the diagnostic strategy relies on integrating clinical and biochemical data with highly sensitive functional imaging, particularly to guide potential surgical management.

Treatment of CHI constitutes a therapeutic emergency, with the primary goal of preventing neurological sequelae associated with severe and prolonged NH. Upon clinical suspicion, urgent management is required without waiting for etiological confirmation. It is imperative to maintain blood glucose levels above 3 mmol/L to protect the developing brain. This necessitates continuous intravenous glucose infusion, often at high rates of 17 to 25 mg/kg/min, justifying the placement of a central venous catheter in the neonate [17]. In cases of symptomatic hypoglycemia, an initial bolus of 10% glucose (2 mL/kg over five minutes) is recommended, followed by continuous infusion at an appropriate rate [1]. When intravenous access is difficult, glucagon can be administered intramuscularly or subcutaneously at doses of 0.5 to 1 mg to rapidly mobilize hepatic glycogen stores [1]. Glucagon may also be given as a continuous infusion at 5 to 20 µg/kg/h. Specific medical treatment primarily relies on diazoxide administration, an activator of KATP channels in β-cells. This drug acts by maintaining these channels in an open state, thereby inhibiting insulin secretion. It is prescribed initially at 5 to 10 mg/kg/day, which can be increased up to 15 to 20 mg/kg/day depending on clinical response. Diazoxide efficacy depends on the functional integrity of KATP channels and is ineffective in cases of inactivating mutations in ABCC8 or KCNJ11 genes. It is recommended to co-administer a diuretic such as hydrochlorothiazide to prevent fluid retention, particularly at treatment initiation. Echocardiography is indicated before starting diazoxide, although a normal initial examination does not exclude the later occurrence of adverse effects. In cases of diazoxide resistance, other agents may be introduced. Octreotide, a short-acting somatostatin analogue, is used as a second-line therapy. It is administered subcutaneously at an initial dose of 15 µg/kg/day, divided into multiple daily injections, with the possibility of gradual dose escalation depending on clinical response [3]. Continuous glucagon infusion also represents a complementary therapeutic option. Emerging treatments such as sirolimus, an mTOR pathway inhibitor, or the GLP-1 receptor antagonist exendin [3] are currently under investigation, but their use remains limited to very specific cases due to uncertain safety profiles.

The duration and efficacy of medical therapy for CHI are determined by multiple factors, including the histopathological subtype (focal or diffuse), the presence or absence of an associated syndromic form, the underlying genetic mutation, and the patient’s clinical and biochemical response. Certain syndromic variants, such as the hyperinsulinism/hyperammonemia syndrome, may exhibit a favorable prognosis despite their diffuse presentation, as they often demonstrate good responsiveness to pharmacological interventions [1]. Medical treatment is maintained until a stable metabolic state is achieved, characterized by normoglycemia without the requirement for intravenous glucose infusion. Given the possibility of spontaneous remission, an annual trial of therapeutic withdrawal under close hospital supervision is recommended [1].

When medical treatment fails, surgery may be considered. In focal forms, identified by [18F]-DOPA positron emission tomography, partial resection limited to the lesion generally allows complete cure. In contrast, for diffuse forms resistant to medical therapy, a subtotal pancreatectomy (95% to 98% of pancreatic volume) may be proposed as a last resort. This intervention is associated with long-term complications, notably insulin-dependent diabetes mellitus and exocrine pancreatic insufficiency, limiting its indication to severe, refractory cases. In our case, surgery was not performed as the patient responded well to medical treatment. Additionally, we were unable to carry out genetic testing and [18F]-DOPA PET scanning due to a lack of resources. Had these investigations been possible, they would have allowed us to determine whether the patient’s hyperinsulinism was of a focal or diffuse form and thereby better guide the surgical management if needed.

Nutritional management constitutes a fundamental pillar of treatment. Feeding should be tailored to ensure glycemic stability and adequate growth. This involves carbohydrate enrichment, increased meal frequency, and, if necessary, oral or enteral glucose-rich supplements. Parental education on glucose monitoring, emergency treatment administration (glucagon), and recognition of hypoglycemia symptoms is essential to optimize outpatient follow-up for patients with CHI.

Children with CHI require close and regular medical follow-up throughout growth due to the potentially complex disease course and associated treatments. This follow-up aims to evaluate treatment efficacy in maintaining glycemic stability, monitor hypoglycemic episode frequency, detect and manage medication side effects, and assess overall development, including growth, neurodevelopment, and metabolic status [3]. The treatments used, particularly diazoxide and octreotide, are not without adverse effects. Diazoxide may cause fluid retention, hypertrichosis, anorexia, bone marrow suppression, and, more rarely, pulmonary hypertension. Therefore, echocardiography is recommended prior to initiation, followed by regular cardiac monitoring. Octreotide may induce growth retardation, gastrointestinal disturbances, and gallstone formation, necessitating careful clinical and laboratory surveillance. The overall prognosis of CHI remains guarded, especially due to the high risk of neurological sequelae associated with severe or prolonged hypoglycemia, which may result in psychomotor delay, cognitive impairments, or epilepsy. Additionally, patients who have undergone subtotal pancreatectomy for diffuse disease face an increased long-term risk of insulin-dependent diabetes and exocrine pancreatic insufficiency.

## Conclusions

CHI is the most frequent and severe cause of persistent hypoglycemia in neonates and infants. Its management relies on rapid clinical recognition, precise biological diagnosis, appropriate use of functional imaging, and tailored therapeutic approaches according to histological type and underlying genetic etiology. Disease progression largely depends on the timeliness of treatment initiation and the effectiveness of glycemic control, which determine neurological prognosis. Close, multidisciplinary monitoring is essential to prevent long-term complications, particularly neurodevelopmental sequelae and metabolic disorders secondary to treatment.
